# Hand Gesture Recognition Using an IR-UWB Radar with an Inception Module-Based Classifier

**DOI:** 10.3390/s20020564

**Published:** 2020-01-20

**Authors:** Shahzad Ahmed, Sung Ho Cho

**Affiliations:** Department of Electronics and Computer Engineering, Hanyang University, 222 Wangsimini-ro, Seongdong-gu, Seoul 04763, Korea; shahzad1@hanyang.ac.kr

**Keywords:** hand gesture recognition, IR-UWB radar, inception module, deep learning, human–computer interaction

## Abstract

The emerging integration of technology in daily lives has increased the need for more convenient methods for human–computer interaction (HCI). Given that the existing HCI approaches exhibit various limitations, hand gesture recognition-based HCI may serve as a more natural mode of man–machine interaction in many situations. Inspired by an inception module-based deep-learning network (GoogLeNet), this paper presents a novel hand gesture recognition technique for impulse-radio ultra-wideband (IR-UWB) radars which demonstrates a higher gesture recognition accuracy. First, methodology to demonstrate radar signals as three-dimensional image patterns is presented and then, the inception module-based variant of GoogLeNet is used to analyze the pattern within the images for the recognition of different hand gestures. The proposed framework is exploited for eight different hand gestures with a promising classification accuracy of 95%. To verify the robustness of the proposed algorithm, multiple human subjects were involved in data acquisition.

## 1. Introduction

In recent years, computing technology has been consolidated in every aspect of our daily lives, and automation is becoming inevitable. As a result, the existing familiar but less natural human–computer interaction (HCI) methods, such as a keyboard and mouse, are becoming a bottleneck [1]. Hand gesture recognition-based HCI provides an intrinsic contactless interface, bringing humans one step closer to a natural mode of interaction. These interactive HCI models have huge potential to be implementable in contactless environments, such as operating rooms [2] and sign language-based communication systems [3]. Conventional HCI approaches mainly utilize optical sensors, speech recognizing sensors, and wearable devices. Optical sensors such as depth cameras are being widely used for motion sensing and gesture recognition [4]. These gesture recognition frameworks are highly accurate but environment-dependent [5]. Highly lit and dark lightning conditions have adverse effects on the overall recognition accuracy. Privacy concern is another downside of camera-based gesture recognition [6]. Speech recognition can also provide an interactive HCI environment. However, the tonal and physical variations, such as background noise, have a drastic effect on the recognition accuracy [7]. In order to deal with these concerns, wearable devices such as gloves may well provide an opportunity. Singh et al. [8] reported an approximate accuracy of 95% for a wearable device-based hand gesture recognition system. However, these additional wearable devices can cause discomfort to users [9], as the users are required to wear certain electromechanical sensing devices. On the other hand, radar sensor-based hand gesture recognition techniques can overcome these limitations. Radar sensors are not affected by lighting conditions, as they can perform well in both highly lit and dark environments. In addition to that, radars do not have any user privacy issues. Moreover, radars provide a contactless environment for capturing gestures where users are not required to wear any additional hardware, unlike wearable sensors. Ultra-wideband (UWB) radar technology uses a transmitter-receiver pair with a widespread frequency spectrum (usually above 500 MHz) and narrow pulse period (on the order of nanoseconds). These types of UWB signaling-based radars are typically referred to as impulse-radio ultra-wideband (IR-UWB) radars [10]. Other types of typical radars used for hand motion sensing and classification are frequency modulated continuous wave (FMCW) radars [11,12] and Doppler radars [13].

The frequency spectrum is turning out to be a scarce resource. To overcome this shortcoming, a UWB communication system is emerging as a candidate solution. In this paper, an IR-UWB radar is used due to its low hardware complexity, high range resolution, and robustness in multi-path environments. IR-UWB radars have found many applications, such as people counting [14] and vital sign detection [15]. Previously, an algorithm based on an IR-UWB radar for hand gesture recognition was proposed by [16]. However, only a three feature-based unsupervised clustering algorithm was used for classification, and the technique only worked well with fewer and highly dynamic gestures. Ren et al. [17] demonstrated an IR-UWB radar-based hand gesture recognition methodology. The gestures were solely classified based on the difference in the final position of performed gestures and may not provide a solution for complex gestures. Khan and Cho [18] proposed a UWB-based hand gesture recognition technique for vehicular applications. Although the accuracy was significantly high, the gesture vocabulary used comprised dynamic and repetitive gestures: the users were required to repeat each gesture motion continuously several times until the final decision was made by the classifier. Ahmed and co-workers proposed a deep-learning-based gesture recognition system to count the number of raised fingers with a significantly high accuracy [19]. However, the algorithm was applicable for a single radar only, and the ergonomics of gestures were not considered.

For recognition and classification problems, usually, the step after data acquisition is feature extraction, followed by classifier training. Several feature extraction techniques exist for IR-UWB signals, where the feature set is prepared as a single dimensional vector [16]. For classification purposes, support vector machines were always preferred over convolutional neural networks (CNNs), until 2012, when Krizhevsky et al. [20] proposed a CNN-based classifier which outperformed the previous classification algorithms. Initially, CNN-based classifiers had a typical structure of stacked layers only, where a convolutional layer was followed by another convolutional layer to form a complete network. Serre and coworkers [21] designed a novel deep network based on the working principle of the visual cortex. In this model, instead of stacking convolutional layers on top of each other to increase the accuracy, a series of gabor filters was applied at the beginning to extract features at different scales. Another similar approach named r-CNN was introduced by Girshick et al. [22], where the input image was divided into several small regions before applying CNN. The derivatives of these networks have found many applications in vision-based human gesture recognition [23]. Inspired by the above-mentioned deep networks, Szegedy and coworkers [24] developed a network with the codename GoogLeNet, which outperformed all existing architectures in ImageNet Large Scale Visual Recognition Competition (ILSVRC), a 2014 image classification challenge. Rather than simply stacking the layers linearly, it is comprised of a novel building block known as an inception module, which contains several small convolutional blocks within the network. Moreover, the inception module-based GoogLeNet classifier turned out to be a solution to overfitting problems. These inception module-based classifiers have found several very recent applications, such as medical image classification [25,26] and handwriting recognition [27]. Previously, researchers paid little attention to evaluating these inception modules for radar signal classification. Wang and co-workers [12] implemented the GoogLeNet classifier to classify gestures using an FMCW radar. In the case of UWB radars, researchers have not treated the acquired gesture data in three-dimensional (3D) way. This paper aims to overcome this challenge for IR-UWB radars. The main idea of implementing inception module-based CNNs for IR-UWB radars is to construct and validate methodology to apply the readily available state-of-the-art deep-learning platforms for UWB radars.

Motivated by the extensive usage of GoogLeNet architecture in the field of image processing [24,25,26,27,28], we present a similar architecture with fewer inception modules than the original GoogLeNet to classify radar signals of different hand gestures. The main objective is to gain a higher gesture recognition accuracy in comparison to that of linear deep-learning frameworks for radars. We used only seven naive inception modules instead of nine and obtained an accuracy slightly higher than the original version of GoogLeNet architecture. In the proposed framework, the received IR-UWB radar signal is treated as a three-dimensional intensity signal comprising red, green, and blue (RGB) values. A gesture vocabulary of eight gestures was selected, and an experimental environment was built to collect several samples for every individual gesture. First, the acquired sample of each gesture was preprocessed and then converted into a 3D intensity pattern. This 3D pattern was further fed to the proposed deep-learning platform as an input. The main contributions of this paper are as follows:

1. We present a novel implementation of the deep-learning algorithm for recognizing hand gestures using IR-UWB radars. To the best of our knowledge, for hand gesture recognition, deep-learning algorithms (based on 3D-CNN architectures), such as inception modules and GoogLeNet, have never been implemented with IR-UWB radars;
2. We present an intuitive scheme to demonstrate hand motions as three-dimensional intensity images for IR-UWB radars;
3. Finally, the integrated framework is tested for a diverse set of gesture vocabulary with two radars. Validation is performed for several samples of each individual gesture. The formulated methodology can further be extended to build any radar signal classifier, regardless of the nature of the application.

The rest of the manuscript is structured as follows: Section 2 deals with the theoretical background, including data acquisition, signal preprocessing, and details on the implemented deep-learning classifier; Section 3 presents an experimental setup based on two radars to validate the theoretical framework presented in Section 2; finally, the experimental results and conclusions are presented in Section 4 and Section 5, respectively.

## 2. Methodology

**Figure 1:**
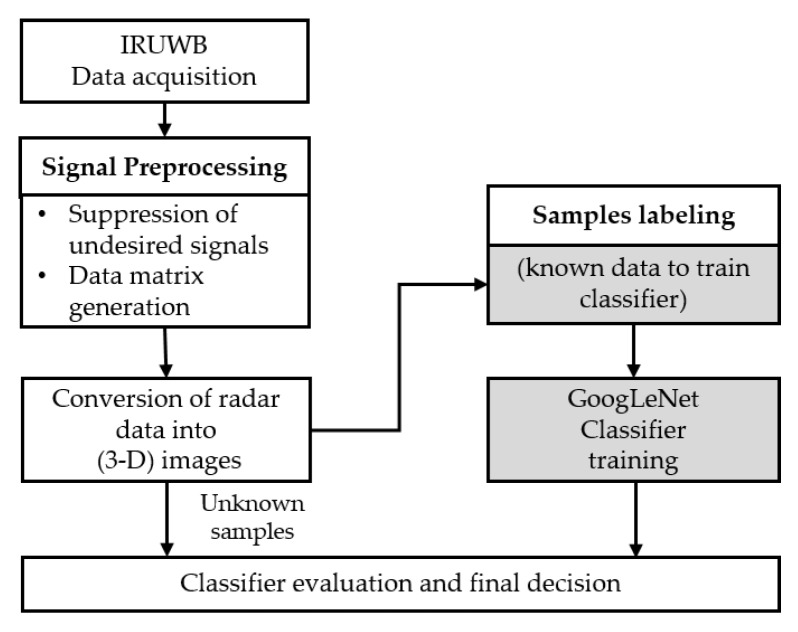
Training and evaluation process of the proposed hand gesture recognition framework for impulse-radio ultra-wideband (IR-UWB) radars.

Figure 1 presents an overall block diagram of the framework proposed in this study. We used IR-UWB radars to collect the gesture data. Every individual sample was first preprocessed and then converted into a 3D image. A classification problem typically consists of a training process and evaluation process. For training purposes, data for each gesture were labeled first, and the classifier was trained to learn the features for each individual label (class). On the other hand, the categorical class of evaluation data was unknown to the classifier, and the classifier could predict the class of data using the pre-trained classifier.

### 2.1. Signal Preprocessing

**Figure 2:**
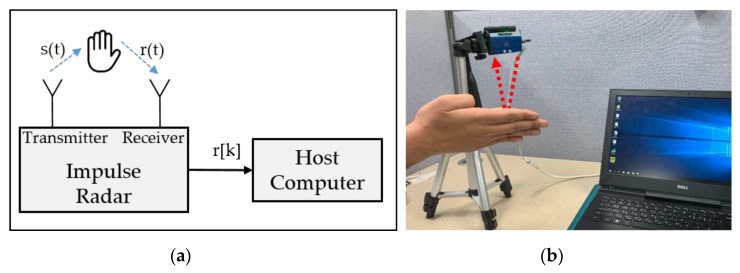
Data acquisition setup: (**a**) Conceptual setup and (**b**) its corresponding actual hardware setup.

In this section, the gesture signal acquisition and preprocessing are demonstrated with a single radar, which is later scaled to the experimental setup consisting of multiple radars. Figure 2a,b represents the respective logical diagram and the corresponding actual setup for data acquisition assembly. The IR-UWB radar transmits a signal comprising a series of pulses with a short time duration and wide frequency spectrum. As shown in Figure 2a, every transmitted continuous pulse s(t) is reflected by the objects present within the operational range of the radar and received at the IR-UWB receiver denoted as r(t). After digitizing *r*(*t*), the signal *r*[*k*] is further analyzed by the host computer, where the pattern recognition task is carried out.

**Figure 3:**
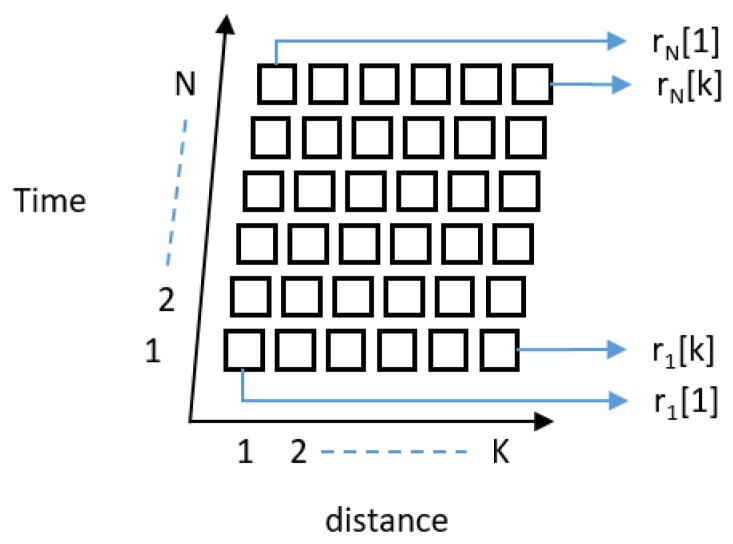
Creation of the IR-UWB radar data matrix comprising *K* fast-time indexes representing each row and *N* slow-time indexes representing each column.

The received signal is stored in the host computer as a 2D data matrix comprising *N* rows and *K* columns, as shown in Figure 3. Here, ‘*N*’ represents the time-sequenced received radar data usually termed the slow-time value, and ‘*K*’ represents the distance of received reflections usually referred to as the fast-time value [14]. The slow-time value is defined by the repetition frequency of the radar, and the fast-time index represents the time of arrival of the signal [14].

In mathematical form, the digitized version of the radar returns *r*[*k*] in two-dimensional form having *N* and *K* slow-time and fast-time indexes, respectively, and can be expressed as

(1) $$\vec{R}=\left[\begin{array}{ccc} r_{N,1} & \cdots & r_{N,K}\\ \vdots & \ddots & \vdots \\ r_{1,1} & \cdots & r_{1,K} \end{array}\right]+{\vec{C}}_{N,K}$$

Here, the term ‘C’ represents the environmental reflections. These are usually the reflections from the static objects present within the operational range of the radar. These unwanted reflections are commonly known as clutter [29].

**Figure 4:**
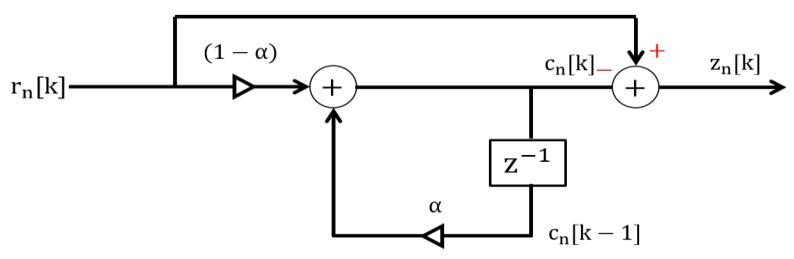
Clutter removal filter with a single-delayed feedback term.

In order to remove the clutter, the received raw signal is passed through a clutter removal filter. For this purpose, we used a simple loop-back filter due to its simple structure and low-computation expense [14,18]. The structure of the clutter removal filter is shown in Figure 4. The filter comprises a recursive structure with a single-delay feedback term. The working principle of the filter shown in Figure 4 can be represented as

(2) $$c_{n}\left[k\right]=\alpha c_{n}\left[k-1\right]+\left(1-\alpha \right)r_{n}\left[k\right].$$

Here, the clutter term $c_{n}\left[k\right]$ is derived by using the previously estimated clutter term $c_{n}\left[k-1\right]$ and the received sample $r_{N}\left[k\right].$ Alpha ‘α’ is the weighting factor ranging between 0 and 1. The estimated clutter is then subtracted from the signal in Equation (1) to obtain a clutter-free signal. We can define the clutter-removed version of the received signal matrix $\vec{Z}$ as

(3) $$\vec{Z}=\vec{R}-{\vec{C}}_{N,K}.$$

### 2.2. Conversion of the Radar Signal into a 3D Image

**Figure 5:**
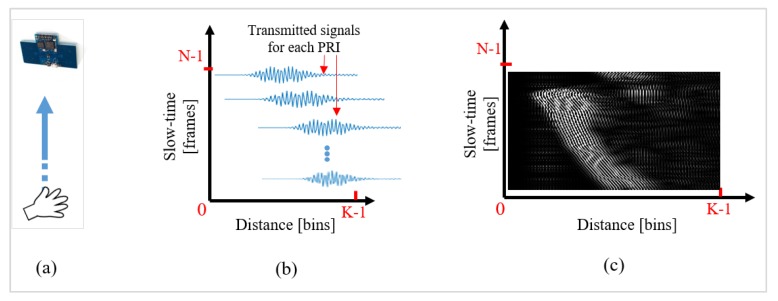
Conversion of the gesture signal to a grayscale pattern. (**a**) Experimental setup showing a hand moving towards the radar; (**b**) respective symbolic representation of individual fast-time signals; (**c**) generated grayscale image.

Since, for every individual sample of a hand gesture, the signal represented by Equation (3) is a two-dimensional matrix, this matrix is first converted into a grayscale (2D) image. Figure 5 represents a detailed operation of converting a gesture signal into a grayscale image. Figure 5a demonstrates a simple scenario of a hand moving towards the radar. The corresponding gesture signal and the grayscale image are shown in Figure 5b,c. For a logical illustration, Figure 5b shows a conceptual two-dimensional signal, whereas Figure 5c represents the actual grayscale image corresponding to the hand movement. Here, fast-time samples (*K*) represent the distance between the performed gesture and radar, whereas the slow-time samples (*N*) represent the duration of the performed gesture. The gesture signal is transformed into a grayscale image by using linear one-to-one mapping of pixels. Radar data are mapped to an integer value between 0 and 255, with 0 representing black and 255 representing white. This normalization process for the above acquired data matrix represented in Equation (4) can be expressed as

(4) $$z_{norm\;\left(i\right)}=\left(255-0\right)\frac{z_{i}-min\left(\vec{Z}\right)}{max\left(\vec{Z}\right)-min\left(\vec{Z}\right)}+0,$$

where $z_{norm\;\left(i\right)}$ refers to the obtained normalized value of the ‘*i*th’ sample *z_i_* within the overall data matrix *Z*. As seen in Figure 5c, as the distance between the hand gesture and radar keeps on decreasing, the high intensity values tend to move towards the left side of the image.

**Figure 6:**
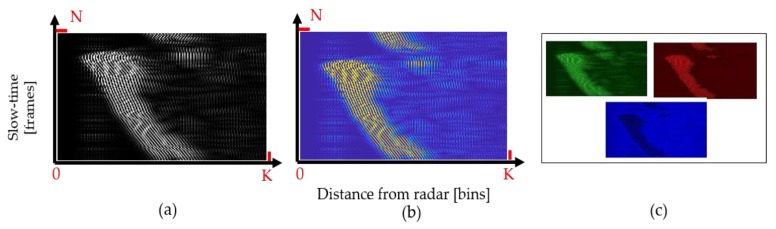
Red, green, and blue (RGB) signal formation for 5.5 s of gesture: (**a**) grayscale radar data; (**b**) corresponding colored image; (**c**) R, G, and B channels of the image in (**b**).

In order to use the 3D CNN-based classifier, the normalized grayscale data are further converted into a colored image. Since the overall motion information is presented by the intensity of the received signal, it will be more convenient to have a wider spread distribution of intensities rather than a grayscale distribution. We used MatLab function ‘ind2rgb8′ to convert the grayscale image into an RGB image. The RGB image corresponding to grayscale representation of the hand gesture signal shown in Figure 5a can be observed in Figure 6b. Additionally, Figure 6c shows the decomposition of the generated colored image into red, green, and blue components.

Algorithm 1 presents the summarized procedure employed to convert the input data into images. By repeating steps 1–6, all the samples of acquired hand gestures are saved as portable network graphic (PNG) images to form a complete dataset. The framework presented to create an image database is implementable with both single and multiple radars and can be used to prepare UWB radar signals for any arbitrary deep-learning algorithm. The case of two radars is further discussed in Section 3 and Section 4.

**Algorithm 1** Transformation of the Radar Signal into a 3D Image
Input: Data Matrix $\vec{R}$Output: RGB image (PNG format)ProcedureStep 1: Collect (2D) radar returns while performing gesture: $\vec{R}$Step 2: within $\vec{R}$, estimate clutter term: $\vec{C}$Step 3: Subtract clutter term from radar returns: $\vec{Z}=\vec{R}-\vec{C}$Step 4: Generate grayscale image of clutter-removed radar returnsStep 5: Convert grayscale image into 3D imageStep 6: Save 3D image in PNG file format.End procedureRepeat 4–9 for all the samples separately.


### 2.3. Feature Extraction and Classification Using an Inception Module-Based CNN

In this paper, the proposed feature extraction network is derived from GoogLeNet, which utilizes inception modules. Next, we will discuss the structural details of each building block.

#### 2.3.1. CNN Architecture

**Figure 7:**
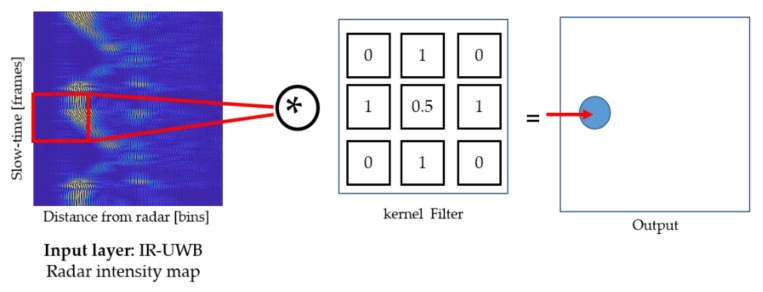
3D convolutional operation for IR-UWB radar data within one hidden layer.

Traditional CNNs are comprised of the following building blocks [20]:

- **Input layer**: Represents the raw input (pixels) in the form of a 2D or 3D matrix;
- **Convolutional layer:** The main objective function of a convolutional layer is to generate a feature map by convolving the input layer with a kernel of a 2D filter with the size ‘a∗b’. This kernel is moved throughout the image to generate the output of the convolutional layer. The process is further demonstrated in Figure 7, where the input pattern is convolved with a 3 × 3 kernel and the resulting output is fed to the forthcoming layer in the architecture;
- **Batch normalization layer:** Deployed after the convolutional layer to further accelerate the training process;
- **Rectified linear unit and max pooling layer:** These layers perform the operation of the activation function and linear down sampling, respectively. Max pooling layers pull the regional maxima from the input, which further reduces the complexity of the network.

#### 2.3.2. Motivation to Use Inception Modules

The above presented layers of CNN architecture (except for the input layer) constitute one hidden layer of a CNN, and several layers are stacked together to form a complete deep network. A linear increment of the number of layers is one straightforward way of increasing the complexity of the network, which can, in turn, result in a higher classification accuracy. However, a linear increment of the number of layers cannot be considered a generalized solution as increasing the number of layers makes the network more vulnerable to overfitting problems [24]. GoogLeNet architecture is an optimized deep-learning network, which utilizes all the available filter sizes to surpass the traditional limitations. Structural-level amendments make the network less prone to overfitting problems [24,26]. In order to achieve a higher gesture recognition accuracy, we used inception modules instead of a linear framework. This novel block is discussed in the next section.

#### 2.3.3. Proposed Classifier for the IR-UWB Radar

**Figure 8:**
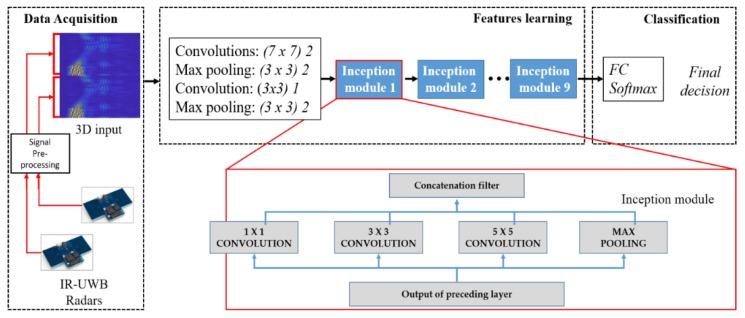
Structure of the proposed classifier, including a data acquisition block, feature extraction block comprising seven inception modules, and classification block.

The confined structure of the implemented deep network for feature extraction and gesture classification is represented in Figure 8. As stated earlier, complex CNN architecture makes a network over-fitted, which results in a huge difference in the training and validation accuracy. For these networks, the training accuracy is much higher than the validation accuracy [24]. To cope with this issue, inspired by the architecture presented by Google, named GoogLeNet, we used inception modules to increase the complexity of the network, rather than linearly stacking the layers. The codename inception is derived from the fact that there exists a network within a network at each hidden layer. We used a basic version of inception modules [24] with different convolutional filters at each hidden layer to extract features. The structure of inception modules is also presented in Figure 8. This deep-learning architecture demonstrated a higher accuracy than the traditional architecture.

Finding the best classifier in terms of accuracy is often subjective, and it can vary based on the nature of the application. Usually, trial-and-error-based search methods can be used to find the optimum size of the network. We tested several possible variants of GoogLeNet architecture for the UWB radar’s gesture data. The accuracy as a function of the number of inception modules was considered, and the one with the highest accuracy is presented in Figure 8. Rather than adding simple layers to increase the complexity, utilizing inception modules can enable us to extract more detailed features of the input data and, in turn, obtain a higher classification accuracy for different gestures. As depicted in Figure 8, our architecture comprises seven basic inception modules, each equipped with three different convolutional layers and a max pooling layer. The overall framework presented in Figure 8 comprises three sub-stages, which are the data-acquisition stage, feature learning stage, and classification stage.

First, the data acquisition block constructs a 3D image input based on the aforementioned algorithm, which serves as an ‘*input layer*’ for our framework. This input is passed to the feature learning block of the classifier, which is comprised of a combination of hidden layers of CNN and a series of seven inception modules. Each individual inception module further consists of three convolutional layers with a filter size of 1 × 1, 3 × 3, and 5 × 5, and a max pooling layer. The outputs of each filter are concatenated together to form the overall output of the corresponding inception module block. The resultant concatenated output serves as an input for the next layer. The inception modules are considered a great milestone in the evolution of CNN classifiers [27]. Inception combinations of higher- and lower-order convolutions capture the features at varying abstraction levels [24]. As stated above, prior to its invention, the CNN’s layers were stacked linearly, with expectations that the new CNN architecture would be more robust and accurate in comparison to the previous one. The inception module was contrived in a way that it should utilize all the available structural variations.

Finally, the gesture classification is performed with a fully connected (FC) layer and softmax decision function. The class having the maximum probability is considered the predicted class at the output of the softmax layer. When a network is trained, this fully connected layer is the final feature vector used for classification or an input to any other network, such as a recurrent neural network (RNN).

## 3. Experimental Setup

### 3.1. Hardware and Software Setup

**Figure 9:**
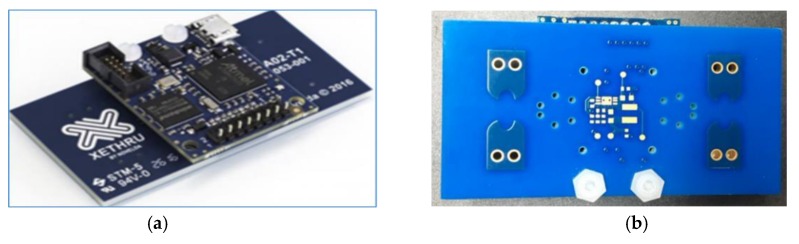
Xethru-X4 radar chip: (**a**) front view and (**b**) back view.

The radar used in this study is the ‘Xethru-X4 radar chip’ designed and manufactured by Novelda (Norway) and has a center frequency of 8.75 GHz. According to federal communications commission (FCC) standards, the unlicensed UWB communication band is limited to a spectrum between 3.1 and 10.6 GHz, and the used radar chip operates within this defined limit. The X4 radar is a high-resolution, low-cost, and low-power solution. The front and back views of the radar chip are shown in Figure 9a,b, respectively. Each radar chip is equipped with a pair of transmit and receive antennas. Further technical specifications of the chip are listed in Table 1. As seen in Table 1, the Xethru-X4 radar can identify moving objects with an accuracy of one millimeter, which makes it possible to differentiate minimal hand movements. Table 1 also suggests that the center frequency and the bandwidth of the radar are in compliance with the FCC standards. The radar was connected to a host computer, and the data were gathered using MatLab.

**Figure 10:**
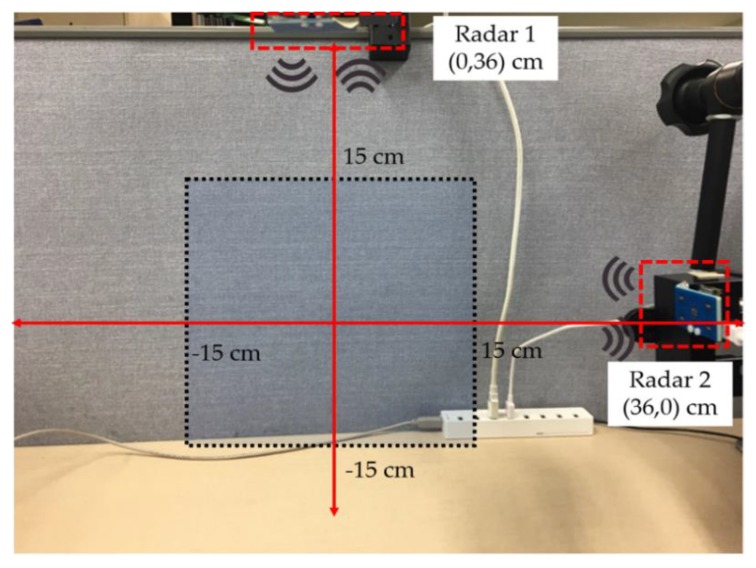
Experimental setup for data acquisition comprised of two radars operating in a mono-static configuration.

Figure 10 shows the experimental environment for data acquisition, with two radars operating in a mono-static configuration where each radar operates independently (as demonstrated in Figure 2). The black highlighted area represents the region where the gestures were performed. The beam width and operational range are the main limiting factors while setting up the experimental environment. Xethru-X4 radars offer a beam width of approximately 65°.

The frame rate of the X4-radar is 20 frames per second (FPS), and a total of 112 slow-time frames were gathered while recording each sample, which means that the duration of each gesture was 5.5 s. The parametric details of signal dimensionality and the hyper-parameter of the implemented classifier are listed in Table 2. For this particular problem, the weighting factor of the clutter removal filter (α) was adjusted to 0.9, solely based on experimentation. The image corresponding to each radar was adjusted to have 112 × 224 pixels for every individual gesture. As a result, collectively, the fused image for two radars had a size of 224 × 224 pixels. While training the network, the learning rate plays an important role as it controls the step-size of the training process. In this study, training was performed using a stochastic gradient descent algorithm with a moderate learning rate of 0.001.

### 3.2. Gesture Vocabulary

**Figure 11:**
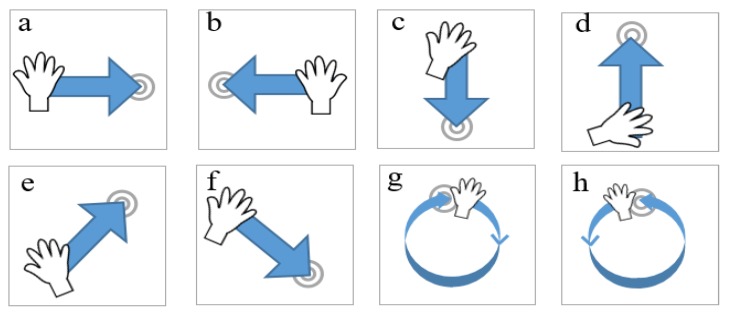
Gesture vocabulary: Eight gestures (**a**–**h**) used to evaluate the classifier.

The above formulated hardware setup was utilized to acquire data against the gestures shown in Figure 11. Eight gestures were carefully selected for a performance evaluation of the hand gesture recognition framework. Figure 11a–h represents the respective left-right swipe (LR-swipe), right-left swipe (RL-swipe), up-down swipe (UD-swipe), down-up swipe (DU-swipe), upward diagonal swipe from left to right (diag-LR-UD-swipe), downward diagonal swipe from left to right (diag-LR-DU swipe), clockwise rotation (CW-rotation), and counterclockwise rotation (CCW rotation). Moreover, we used three different human subjects to gather training data to avoid biasness in training and testing samples. In total, 100 samples were collected for each gesture, and 80% of the data were used for training purposes, whereas 20% were used for evaluating the trained classifier.

## 4. Experimental Results

### 4.1. Single Dimensional Clutter Removal Results

**Figure 12:**
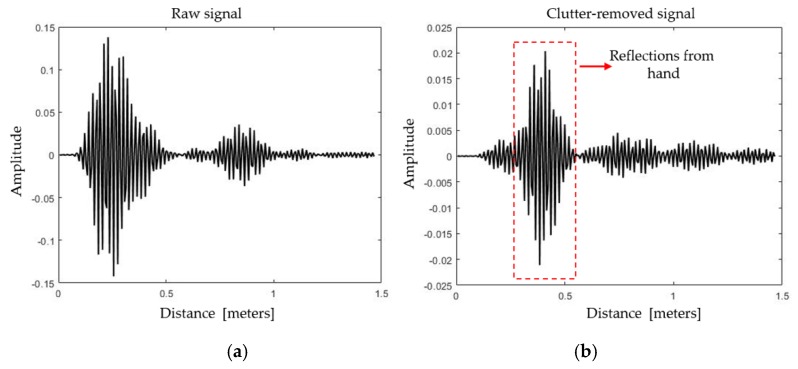
Single dimensional clutter removal operation: (**a**) input and (**b**) output of the clutter removal filter.

Figure 12a,b represents the respective intermediate output of the deployed clutter removal filter for a single fast-time repetition interval while performing the gesture. In Figure 12, the received signal voltage is plotted against the distance in meters. It can be observed that the clutter effect in the signal at the input of the filter has greatly been suppressed by the filter, and a clutter-free signal can be seen at the output. At the output of the clutter removal filter, the gesture information, centered approximately at 0.4 m, is visibly enhanced. For the Xethru-X4 radar, a distance of one meter comprises 156 fast-time bins (samples). As shown in Figure 12b, a reflection located at an approximate distance of 0.4 m corresponds to reflections centered at approximately the 60th bin. The expected decrease in the signal at the output of the clutter-removal filter can be amplified. The amplitude of the received signal depends on the radar cross section (RCS) as well. A higher RCS results in a high amplitude of the signal at the output of the clutter removal filter.

### 4.2. 2D Clutter Removal Results

**Figure 13:**
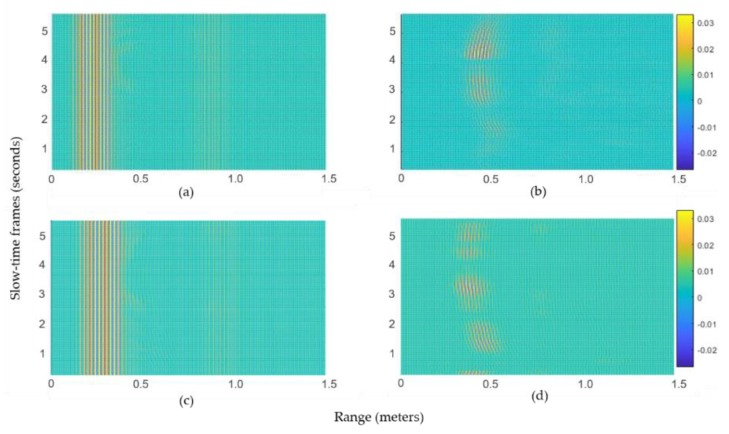
Patterns of recorded gestures for a 5.5 s duration: (**a**) Gesture-1 before and (**b**) after the clutter removal operation. (**c**) Gesture-2 before and (**d**) after the clutter removal operation.

The clutter removal process represented by Equations (2) and (3) is demonstrated here in two dimensions. For simplicity, only the data from radar-1 are discussed. Figure 13a,b represents the signal with and without clutter, respectively, for gesture 1 (LR-swipe), whereas Figure 13c,d represents similar results for gesture 2 (RL-swipe). In the presence of clutter, the respective motion patterns are not visible in Figure 13a,c, whereas these patterns became visible in Figure 13b,d. Note that the clutter discussed here indicates the reflections from static objects.

### 4.3. Image Pattern Analysis of Recorded Hand Gestures

**Figure 14:**
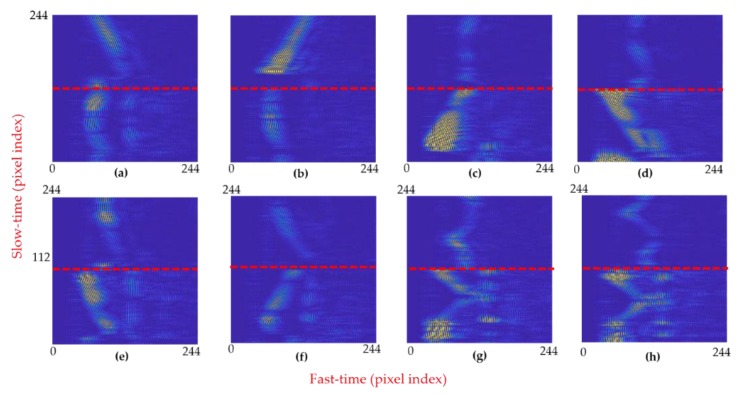
Image patterns of both radars for eight selected gestures. The red line separates data against radar-1 and radar-2.

Next, the output of all the radars was concatenated together to form a single concatenated image. For the gesture vocabulary presented in Figure 11a–h, the corresponding image patterns are shown in Figure 14a–h. The red highlighted line in Figure 14 marks the boundary between the signals of each radar: The part of the image above the red line corresponds to radar-2, whereas the part of the image below the red line is the pattern against radar 1. In terms of slow-time frames, for images corresponding to every individual sample of performed gestures, the slow-time frames ranging from 1 to 112 present the signal acquired by radar-1, and the slow-time frames ranging from 113 to 224 present the signal acquired by radar-2. Since the output images of both radars are concatenated vertically, the overall size of individual images presented in Figure 14 is 224 × 224 pixels. The width and the height of images are controlled directly by adjusting the fast-time and slow-time values, respectively. A complete gesture motion should be recorded within the designated image frame to avoid errors.

While analyzing the images generated by radar-1 in Figure 14, i.e., below the red line, it can be seen that for LR- and RL-swipe presented in 14a,b, the image pattern is similar in both cases. We can say that the motion information expressed as variation in slow-time appears to be constant with respect to the vertical axis. On the other hand, for the upward and downward swipe presented in Figure 14c,d, a diagonally moving intensity pattern is observed. For the clockwise and anticlockwise circular motions depicted in Figure 14g,h, the image shows a circular pattern. By adding multiple radars in the experimental setup, we can create highly varying patterns for different gestures that will strengthen the overall classification process.

The images shown in Figure 14a–h serve as an input for the feature extraction block. The patterns demonstrated in Figure 14 also suggest that the addition of more radars creates highly varying patterns for each of the gestures. As stated above, in the case of LR-swipe and RL-swipe, the image created against the data of radar-1 is similar in both cases, which makes it hard for the classifier to distinguish them. On the contrary, the upper-half of Figure 14a,b representing the output of radar-2 for RL- and LR-swipe, displays a distinguishable pattern. The patterns against LR- and RL-swipe gestures for radar-2 in Figure 14a,b have positive and negative slopes, respectively. Similar kinds of trends can be seen for the patterns of remaining gestures. As a result, the fused images exhibit a clear difference in the patterns.

### 4.4. Analysis of Variations in Patterns of the Same Gestures

**Figure 15:**
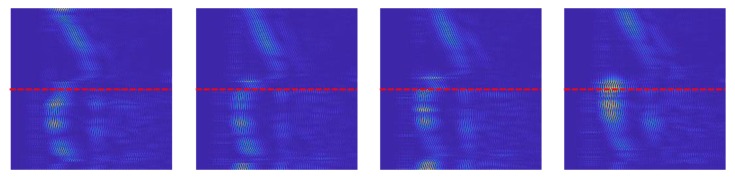
Inter-sample pattern analysis: Pattern of images for four different samples of the same gesture (LR-swipe).

Figure 15 represents the variation in the generated images for the same gesture. Figure 15 shows four different samples of the LR-swipe gesture for the same human volunteer. As observed in Figure 15, there exists a slight difference in the intensities of each sample. Since human volunteers performed the gestures without fixing the orientation of their hand, a change in the reflective area of the hand directly affects the intensity of the received signal. However, the overall shape of the pattern for the LR-swipe gesture remains similar. No restriction in the speed of the performing gesture was imposed on the volunteers to make the algorithm robust.

### 4.5. Classification Accuracy

**Figure 16:**
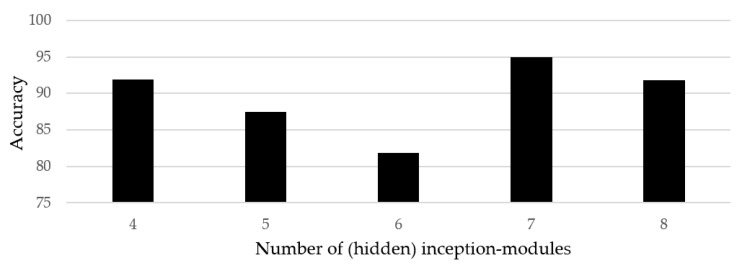
Results of classification accuracy for different numbers of inception-modules.

We performed different structural variations while designing the deep-learning architecture for classifying hand gestures. In order to find the optimized network, several experiments with different numbers of inception modules were performed. The accuracy as a function of the number of inception modules is shown in Figure 16. Here, the vertical axis represents the accuracy, whereas the horizontal axis represents the classifiers with different numbers of inception modules. It was observed that the networks with 4, 5, 6, and 8 inception modules demonstrated an approximate accuracy of 91.87%, 87.5%, 81.87%, and 91.87%, respectively. In addition to that, fewer than four inception modules yielded an accuracy of less than 50% as the network was too shallow and was not able to extract the features properly. On the other hand, the classifier with seven inception modules stood amongst the others, with an average accuracy of 95%. Better convergence of the acquired dataset was observed against the classifier structure with seven inception modules, as presented in Section 2.3.

**Figure 17:**
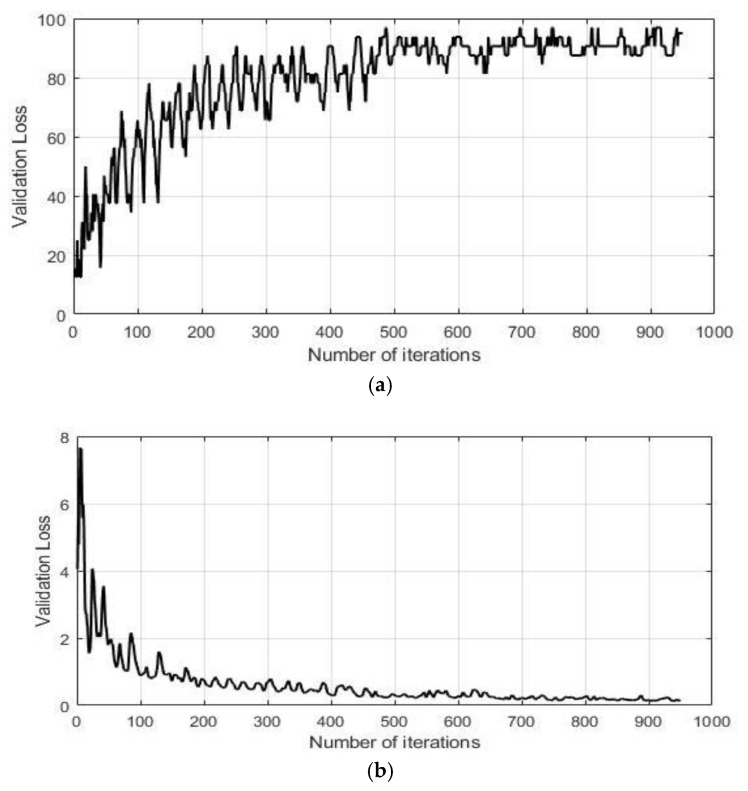
Learning trend of GoogLeNet for the IR-UWB radar with 1920 epochs: (**a**) validation accuracy in percentage and (**b**) validation loss.

The classification accuracy and classification loss of the proposed classifier for the IR-UWB radar are presented in Figure 17a,b. The experiment was performed several times for validation of the results, with random splitting of the data samples for training and evaluation purposes. The final accuracy for the selected gesture vocabulary turned out to be 95%. The learning trend is shown in Figure 17a, where the network was trained for 950 iterations. Figure 17b represents the average validation loss. With fewer exceptions, validation loss shows a decreasing trend, with a maximum value of 7.5 in the beginning and 0.11 at the end. Unlike accuracy, validation loss is expressed as a constant representing the summation of error occurring for every individual iteration. For deep networks exhibiting large structural variations, more than one optimum solution may exist, which in turn causes the accuracy curve to fluctuate before settling down. A similar effect can be seen in Figure 17, where both the accuracy and validation loss curves show fluctuations before reaching final values for the proposed network.

Table 3 presents the confusion matrix of the proposed gesture recognition framework. Here, the columns present the original gesture class and the rows present their predicted gesture class. The classification accuracy of each gesture is presented diagonally in green, and the error values are shown red. As we can see, the swiping along the horizontal and vertical axes shows a higher accuracy in comparison to the other gestures as it generates highly varying patterns in comparison to the remaining gestures. The remaining hand gestures exhibit a slightly higher error rate in comparison to the swiping gestures. It is observable that a straight to and fro motion of the hand in front of a radar (as in the case of the first four gestures) creates a more distinctive pattern in comparison to other complex motions.

### 4.6. Comparison with Existing Techniques

We compared the results of the proposed algorithm with several existing algorithms for performance evaluation, and the results are presented in Table 4. First, we compared the accuracy with that of original GoogLeNet and with a 3D-CNN architecture. The original GoogLeNet architecture was trained using the acquired radar data. The CNN classifier with seven layers and the original GoogLeNet classifier that comprised nine inception modules were trained and evaluated for the same dataset. For the case of CNN architecture, the size of the convolution kernel for each layer of the CNN was 8, 16, 32, 64,128, 256, and 512, with a 3 × 3 filter size. These two classifiers yielded an accuracy of 91.25% and 93.75%, respectively, as represented in the second column of Table 4.

In order to compare the accuracy with literature, recent hand gesture recognition frameworks presented by Skaria et al. (2019) [13] and Kim et al. (2016) [11] were also evaluated in the acquired gesture dataset. Both these algorithms also utilize deep-learning architecture-based features extraction techniques, having only one filter at each hidden layer. However, in our case, several filters are used simultaneously at each hidden layer for feature learning, and their output is concatenated at the end using a concatenation filter. The accuracy of these two algorithms for the above-mentioned experimental setup is presented in the second column of Table 4.

To further support the effectiveness of the proposed system, classification results for data against a single radar only (i.e., radar 1) were also gathered. In this case, the training data and evaluation data acquired with radar 1 were fed into all the three gesture recognition systems as the input. The objective of performing these additional experimentations with a single radar was to ensure that the proposed system can work well under different conditions. It can be seen that the overall accuracy of all the presented systems is considerably higher. However, it can also be seen that the proposed classifier with seven inception modules reported a higher accuracy in comparison to the other classifiers.

## 5. Discussion and Conclusions

In this study, we have demonstrated a novel hand gesture framework based on GoogLeNet and the inception modules. Detailed implementation of a hand gesture recognition system using an IR-UWB radar is presented. First, the IR-UWB radar signal is converted into a grayscale image, and then the grayscale image is mapped into a (3D) RGB image. Data samples are gathered for each individual gesture, which are then divided into training and test datasets. GoogLeNet architecture was used for feature extraction and classification purposes, which leveraged the need for a separate feature vector. Preliminary experiments cross-verified the capability and potential applicability of the proposed hand gesture recognition system for UWB radars. Three volunteers were involved in the experimentation process to make the classifier robust against hand sizes. In addition to that, no restriction was imposed on the human subjects, other than being in the confined area and the designated timeframe.

In this study, the designed gesture vocabulary was two-dimensional, i.e., the motion in the third axis was not considered since we used a highly directional antenna with a beam width of 65°. Furthermore, several patterns of the final images showed the presence of outliers in the acquired data. This may occur due to the change in RCS while performing the gesture. Further research is required to overcome this challenge in a real-time scenario. In addition to that, the gestures were acquired manually within a fixed time duration. However, in order to make data acquisition more realistic, a separate algorithm is required to recognize and separate gesture and non-gesture signals. Confining the motion of hand within the designated area and time-frame is another challenge. The human volunteers involved were advised to perform gesture motions within the highlighted area.

As future work, we will design a real-time version for embedded system implementation to construct a standalone hardware and software solution.

## Figures and Tables

**Table 1:** Specifications of a single IR-UWB radar chip.

Technical Parameter	Specification
Accuracy	~1 mm
Center frequency	8.748 GHz
Frame rate (slow-time sampling rate)	20 frames/s
Bandwidth (–10 dB)	2 GHz
Pulse repetition frequency	40.5 MHz
Antenna beam width	65°
Number of antennas	2 pairs of transmitters and receivers

**Table 2:** Signal specification and hyper parameters of the classifier.

Parameter	Value
Clutter removal filter coefficient (alpha)	0.9
Single radar data size (height × width)	112 × 224 pixels
Size of image with two radars (height × width)	224 × 224 pixels
Learning rate for GoogLeNet	0.001
Optimizer	Stochastic gradient descent.
Learning iterations	950

**Table 3:** Classification accuracy of each gesture. Intensity of green and red colors represent the intensity of classification accuracy and error respectively.

		Original Gesture Class							
		LR Swipe	RL Swipe	UD Swipe	DU Swipe	Diag-LR-UD Swipe	Diag-LR-DU SWIPE	Cw Rotation	CCW Rotation
**Predicted gestures class**	LR-swipe	100	0	0	0	5	0	0	0
	RL-swipe	0	95	0	0	0	0	0	0
	UD-swipe	0	0	95	0	0	0	0	0
	DU-swipe	0	0	0	100	0	0	0	0
	Diag-LR-UD-swipe	0	0	5	0	90	10	0	0
	Diag-LR-DU-swipe	0	0	0	0	5	90	0	0
	CW-rotation	0	5	0	0	0	0	95	5
	CCW rotation	0	0	0	0	0	0	5	90

**Table 4:** Accuracy comparison of the proposed technique with the literature.

Classifier	Accuracy with Two Radars (Figure 10)	Accuracy with a Single Radar
Proposed	95.00%	92.50%
3D-CNN	91.25%	90.62%
GoogLeNet	93.75%	91.25%
Skaria et al. [13]	91.87%	87.50%
Kim et al. [11]	86.87%	82.50%

## References

[1] Yeo H.S., Lee B.G., Lim H. (2015). Hand tracking and gesture recognition system for human-computer interaction using low-cost hardware. Multimed. Tools Appl..

[2] Wachs J.P., Stern H.I., Edan Y., Gillam M., Handler J., Feied C., Smith M. (2008). A gesture-based tool for sterile browsing of radiology images. J. Am. Med. Inform. Assoc..

[3] Kumar P., Gauba H., Roy P.P., Dogra D.P. (2017). Coupled HMM-based multi-sensor data fusion for sign language recognition. Pattern Recognit. Lett..

[4] Kumar P., Jaiswal A., Deepak B., Reddy G.R.M. (2018). Hand Gesture-Based Stable PowerPoint Presentation Using Kinect. Progress in Intelligent Computing Techniques: Theory, Practice, and Applications.

[5] Li K., Jin Y., Akram M.W., Han R., Chen J. (2019). Facial expression recognition with convolutional neural networks via a new face cropping and rotation strategy. Vis. Comput..

[6] Schiff J., Meingast M., Mulligan D.K., Sastry S., Goldberg K. (2009). Respectful cameras: Detecting visual markers in real-time to address privacy concerns. Protecting Privacy in Video Surveillance.

[7] Gifford R.H., Noble J.H., Camarata S.M., Sunderhaus L.W., Dwyer R.T., Dawant B.M., Labadie R.F. (2018). The relationship between spectral modulation detection and speech recognition: Adult versus pediatric cochlear implant recipients. Trends Hear..

[8] Singh G., Nelson A., Robucci R., Patel C., Banerjee N. Inviz: Low-power personalized gesture recognition using wearable textile capacitive sensor arrays. Proceedings of the 2015 IEEE International Conference on Pervasive Computing and Communications (PerCom).

[9] Rautaray S.S., Agrawal A. (2015). Vision based hand gesture recognition for human computer interaction: A survey. Artif. Intell. Rev..

[10] Yarovoy A.G., Ligthart L.P., Matuzas J., Levitas B. (2006). UWB radar for human being detection. IEEE Aerosp. Electron. Syst. Mag..

[11] Kim Y., Toomajian B. (2016). Hand gesture recognition using micro-Doppler signatures with convolutional neural network. IEEE Access.

[12] Wang Y., Wang S., Zhou M., Jiang Q., Tian Z. (2019). TS-I3D based Hand Gesture Recognition Method with Radar Sensor. IEEE Access.

[13] Skaria S., Al-Hourani A., Lech M., Evans R.J. (2019). Hand-Gesture Recognition Using Two-Antenna Doppler Radar with Deep Convolutional Neural Networks. IEEE Sens. J..

[14] Choi J.W., Quan X., Cho S.H. (2017). Bi-directional passing people counting system based on IR-UWB radar sensors. IEEE Internet Things J..

[15] Lee Y., Choi J.W., Cho S.H. Vital sign quality assessment based on IR-UWB radar sensor. Proceedings of the 2017 International Conference on Information and Communication Technology Convergence (ICTC).

[16] Khan F., Leem S., Cho S. (2017). Hand-based gesture recognition for vehicular applications using IR-UWB radar. Sensors.

[17] Ren N., Quan X., Cho S.H. (2016). Algorithm for gesture recognition using an IR-UWB radar sensor. J. Comput. Commun..

[18] Khan F., Cho S.H. Hand based Gesture Recognition inside a car through IR-UWB Radar. Proceedings of the 2017 International Conference on Electronics, Information, and Communication.

[19] Ahmed S., Khan F., Ghaffar A., Hussain F., Cho S.H. (2019). Finger-Counting-Based Gesture Recognition within Cars Using Impulse Radar with Convolutional Neural Network. Sensors.

[20] Krizhevsky A., Sutskever I., Hinton G.E. (2012). Imagenet classification with deep convolutional neural networks. Adv. Neural Inf. Process. Syst..

[21] Serre T., Wolf L., Bileschi S., Riesenhuber M., Poggio T. (2007). Robust object recognition with cortex-like mechanisms. IEEE Trans. Pattern Anal. Mach. Intell..

[22] Girshick R., Donahue J., Darrell T., Malik J. Rich feature hierarchies for accurate object detection and semantic segmentation. Proceedings of the IEEE Conference on Computer Vision and Pattern Recognition.

[23] Noori F.M., Wallace B., Uddin M.Z., Torresen J. (2019). A Robust Human Activity Recognition Approach Using OpenPose, Motion Features, and Deep Recurrent Neural Network. Scandinavian Conference on Image Analysis.

[24] Szegedy C., Liu W., Jia Y., Sermanet P., Reed S., Anguelov D., Rabinovich A. Going deeper with convolutions. Proceedings of the IEEE Conference on Computer Vision and Pattern Recognition.

[25] Bai J., Jiang H., Li S., Ma X. (2019). NHL Pathological Image Classification Based on Hierarchical Local Information and GoogLeNet-Based Representations. BioMed Res. Int..

[26] Fang T. A Novel Computer-Aided Lung Cancer Detection Method Based on Transfer Learning from GoogLeNet and Median Intensity Projections. Proceedings of the 2018 IEEE International Conference on Computer and Communication Engineering Technology (CCET).

[27] Zhong Z., Jin L., Xie Z. High performance offline handwritten chinese character recognition using googlenet and directional feature maps. Proceedings of the 2015 13th International Conference on Document Analysis and Recognition (ICDAR).

[28] Khan R.U., Zhang X., Kumar R. (2019). Analysis of ResNet and GoogleNet models for malware detection. J. Comput. Virol. Hacking Tech..

[29] Chernyak V.S. (2018). Fundamentals of Multisite Radar Systems: Multistatic Radars and Multistatic Radar Systems.

